# Mortality and morbidity after colorectal cancer resection surgery in elderly patients: a retrospective population-based study in Sweden

**DOI:** 10.1186/s12957-024-03316-6

**Published:** 2024-01-22

**Authors:** Maria Normann, Niklas Ekerstad, Eva Angenete, Mattias Prytz

**Affiliations:** 1 Department of Surgery, Institute of Clinical Sciences, Sahlgrenska Academy, University of Gothenburg, Gothenburg, Sweden; 2 Department of Surgery, Region Västra Götaland, NU-Hospital Group, Trollhättan, Sweden; 3 Department of Health, Medicine and Caring Sciences, Linköping University, Linköping, Sweden; 4 Department of Research and Development, Region Västra Götaland, NU-Hospital Group, Trollhättan, Sweden; 5 Department of Surgery, SSORG — Scandinavian Surgical Outcomes Research Group, Institute of Clinical Sciences, Sahlgrenska Academy, University of Gothenburg, Gothenburg, Sweden; 6 Department of Surgery, Region Västra Götaland, Sahlgrenska University Hospital, Gothenburg, Sweden

**Keywords:** Colorectal neoplasm, Surgery, Elderly, Frailty

## Abstract

**Background:**

Colorectal cancer is primarily a condition of older adults, and surgery is the cornerstone of treatment. As life expectancy is increasing and surgical techniques and perioperative care are developing, curative surgery is often conducted even in ageing populations. However, the risk of morbidity, functional decline, and mortality following colorectal cancer resection surgery are known to increase with increasing age. This study aims to describe real-world data about postoperative mortality and morbidity after resection surgery for colorectal cancer in the elderly (≥ 70 years) compared to younger patients (< 70 years), in a Swedish setting.

**Methods:**

A cohort study including all patients diagnosed with colorectal cancer in a Swedish region of 1.7 million inhabitants between January 2016 and May 2020. Patients were identified through the Swedish Colorectal Cancer Registry, and all baseline and outcome variables were extracted from the registry. The following outcome measures were compared between the two age groups: 90-day mortality rates, postoperative complications, postoperative intensive care, reoperations, readmissions, and 1-year mortality. To adjust the analyses for baseline confounders in the comparison of the outcome variables, the following methods were used: marginal matching, calliper (ID matching), and logistic regression adjusted for baseline confounders.

**Results:**

The cohort consisted of 5246 patients, of which 3849 (73%) underwent resection surgery. Patients that underwent resection surgery were significantly younger than those who did not (mean ± SD, 70.9 ± 11.4 years vs 73.7 ± 12.8 years, *p* < 0.001). Multivariable analyses revealed that both 90-day and 1-year mortality rates were higher in older patients that underwent resection surgery (90-day mortality *OR* 2.12 [95% *CI* 1.26–3.59], *p* < 0.005). However, there were no significant differences in postoperative intensive care, postoperative complications, reoperations, or readmissions.

**Conclusion:**

Elderly patients suffer increased postoperative mortality after resection surgery for colorectal cancer compared to younger individuals. Given the growing elderly population that will continue to require surgery for colorectal cancer, more efficient ways of determining and handling individual risk for older adults need to be implemented in clinical practice.

## Introduction

With a median age of 70 years at diagnosis, colorectal cancer (CRC) incidence increases with age and is a leading cause of cancer-related mortality in the older adults [1–4]. As average life expectancy continuously increases, CRC incidence rates in older patients are likely to follow [5, 6]. Older patients, especially those with chronic and severe concomitant diseases or late-stage disease, are generally less likely to receive surgical intervention or adjuvant chemotherapy compared to younger populations [1, 5–9]. Therefore, there is a risk that elderly patients are undertreated, which will have an adverse effect on prognosis [1, 5, 8] for this patient group.

Previous studies have described that 5-year cancer-specific survival rates for patients with CRC are not age related [1, 4, 10–14]. Nevertheless, elderly patients are known to suffer a high risk of perioperative death following CRC surgery [12, 15–17]. Furthermore, 30-day mortality rates, length of hospital stay, and hospital costs are higher for the older patients [6, 18]. Other established risk factors for a more unfavourable outcome following surgery are a higher American Society of Anaesthesiology (ASA) grade [6], urgent surgery [17, 19], and advanced tumour stage [20]. Despite receiving elective colorectal cancer surgery less frequently, the elderly more often undergoes urgent surgery, as well as non-restorative surgery [6, 12, 17, 18, 21–23]. Compared to younger individuals, urgent surgery in the older patient encompasses an even higher risk of postoperative complications and mortality [12, 24]. In addition, the need of postoperative intensive care further increases the risk of complications [6, 25].

It is acknowledged that the patient’s overall status, including comorbidities, disease stage at presentation, and general physical abilities, plays a more important role on patient outcome after colorectal surgery than chronological age [1, 6]. When treating an older patient at high risk of complications, the choice of treatment needs to be carefully planned and individualised so that it is both effective and safe [1, 4, 17]. It is of importance to recognise the increasing age in the population and incorporate this knowledge in treatment decisions [8, 18]. The continuous demographic development results in a growing elderly population, with increasing need of colorectal cancer surgery. This implies the need of up-to-date knowledge of post-operative results, to further optimise and individualise treatment. This study aims to describe the contemporary situation regarding differences in post-operative outcome, comparing older and younger patients, in a Swedish setting. The primary aim of the present study was to examine the frequency of post-operative mortality and complications following colorectal cancer surgery in Sweden stratified by age, i.e., elderly (≥ 70 years) and younger (< 70 years) populations. The hypothesis was that elderly patients suffer from higher post-operative mortality and more post-operative complications.

## Methods

### Study design and population

This is a registry-based observational study including all individuals diagnosed with colorectal cancer in the Swedish County Region Västra Götaland during January 1, 2016, to May 31, 2020, registered in the Swedish Colorectal Cancer Registry (SCRCR). Region Västra Götaland has approximately 1.7 million residents with six county hospitals and one university hospital [26]. Both baseline and outcome variables were retrospectively obtained from SCRCR, which is a nationwide registry with high coverage (> 98%) of patients with adenocarcinoma of colon and rectum [27]. Data extraction from SCRCR was done in June 2021, and the inclusion period was set to end on May 31, 2020, to ensure correct 1-year mortality data. The outcomes of mortality and morbidity were analysed in relation to one surgical procedure, and if two or more synchronous tumours were treated at the same procedure, only the tumour with the highest clinical staging was kept in the analysis. There were no other exclusion criteria. All participants were older than 18 years of age.

### Variables and outcomes

Patients were grouped in two cohorts based on age at diagnosis (≥ 70 vs < 70 years). The median age of diagnosis of colon versus rectal cancer in Sweden during the years 2016–2020 was 74 (colon) and 71 (rectal) [28, 29]. The cutoff 70 years was chosen as it is clinically relevant and a distinction that is commonly used when comparing older adults to younger individuals. The following baseline variables were extracted for all subjects: age, sex, date of diagnosis, tumour location and stage according to TNM, American Society of Anaesthesiologists (ASA) classification, date of surgery, intraoperative bleeding, and elective versus urgent surgery. The primary outcome measure was 90-day overall survival after resection surgery. Secondary outcomes were post-operative surgical complications and overall complications, reoperations, intensive care unit (ICU) care, readmissions within 30-day post-surgery, and all-cause 1-year mortality.

### Statistical analysis

To compare the two groups, three methods were used: the best marginal distribution matched group, the best caliper-based ID-matched group, and logistic regression adjusted for the confounders. Marginal matching and caliper matching were performed to balance the distribution of the known baseline variables (sex, ASA classification, tumour location, TNM stage, elective vs urgent procedure, and intraoperative bleeding) between cohorts. The matching was done blinded to the outcome variables.

In the matched groups, categorical outcome variables were compared using Fisher’s exact test and presented as mean percent differences (MPD) with 95% confidence interval (CI) together with effect size and *p*-value. In the adjusted analyses on all subjects, multivariable logistic regression adjusted for baseline factors was performed and presented as odds ratio (OR) with 95% CI. Comparison of time to death between the two cohorts was calculated using Cox proportional hazard regression models adjusted för baseline confounders. The results were given as hazard ratio (HR) with 95% CI. The survival analyses were performed on all subjects and of the matched groups respectively. All tests were two-tailed and conducted at 0.05 significance level. The statistical analyses were performed using SAS Version 9.4 (SAS Institute, Cary, NC, USA).

#### Matching methods

Marginal distribution matching is a group-level matching. The participants in the older subgroup were selected one by one, choosing individuals with similar mean values of the baseline confounding variables as the younger subgroup. This was done until no more controls could be included without making the groups too dissimilar.

Caliper matching is an ID-level matching. For each subject in the older subgroup, a matching control subject in the younger cohort was identified through baseline variable values.

## Results

### Patient characteristics

**Fig. 1:**
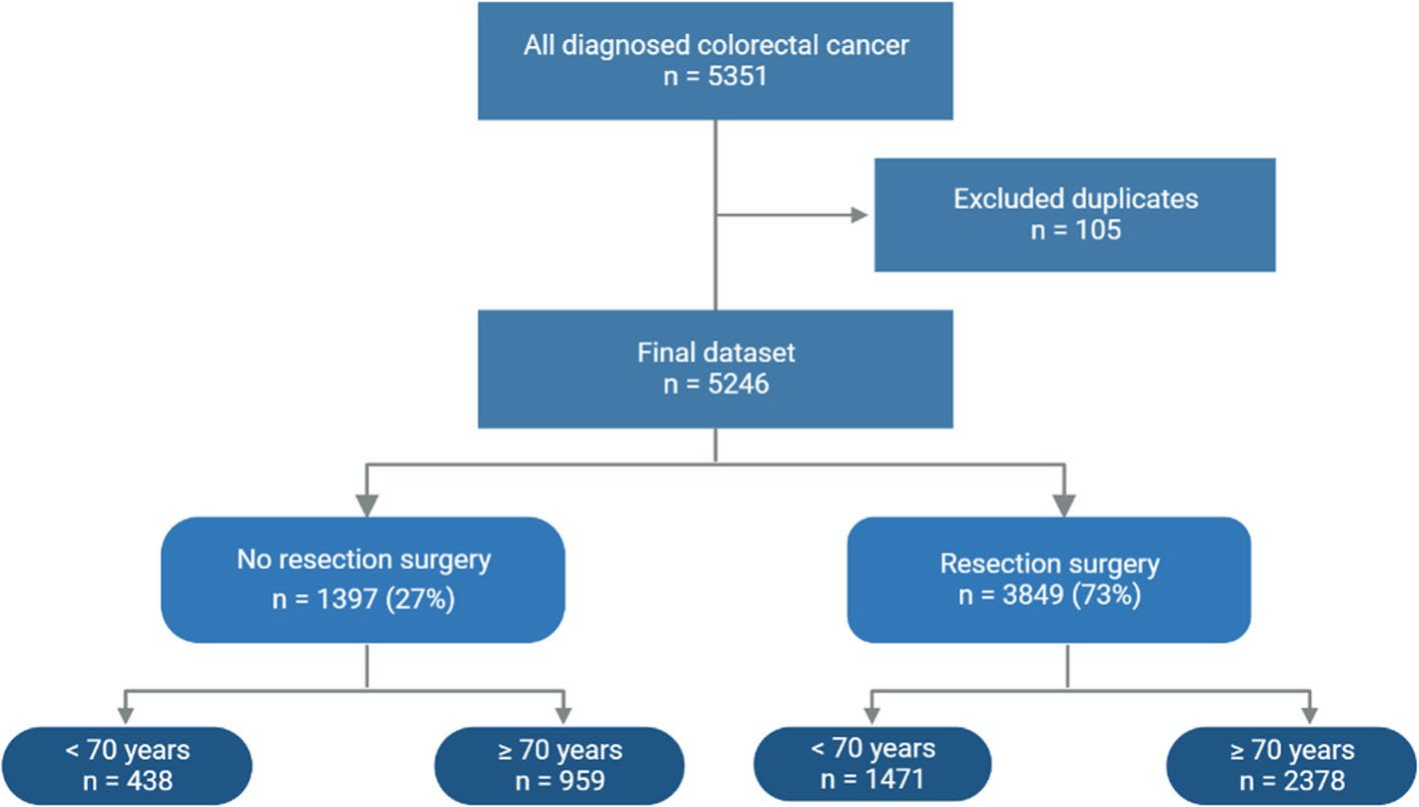
Description of study population, all patients diagnosed with colorectal cancer in Region Västra Götaland during the period January 1, 2016, to May 31, 2020, treatment, and age distribution. Data retrospectively obtained from the Swedish Colorectal Cancer Registry (SCRCR)

In total, 5351 patients were diagnosed with colorectal cancer in Region Västra Götaland, between January 2016 and May 2020. After exclusion of duplicates, the dataset included 5246 patients, of which 3849 (73%) underwent resection surgery. Patients not receiving resection surgery were significantly older than those in the resection surgery group (mean ± SD, 73.7 ± 12.8 years vs 70.9 ± 11.4 years, *p* < 0.001). Stratification of the dataset by age (< 70 and ≥ 70 years) showed that 2378/3849 patients (62%) that underwent resection surgery were aged ≥ 70 years, while 959/1397 patients (69%) not receiving resection surgery were aged ≥ 70 years (Fig. 1).

Baseline characteristics of the resection surgery group are described in Table 1. The two age groups had similar distributions of most baseline variables, but there was a significant difference in ASA classification and tumour location. The older cohort had higher ASA grade (ASA III, 37.3% vs 16.8%; *p* < 0.001), and there were less patients with rectal cancer in the older group. Further sensitivity analyses were made using marginal matching and caliper matching on original variables, to balance the distribution of baseline variables known to affect death after colorectal cancer resection surgery. After matching, the groups were comparable (Table 1).

**Table 1:** Patient characteristics for the resection surgery group, stratified by age. Baseline variables presented for all subjects, after marginal matching and after caliper matching respectively. Subjects with missing data for any of the variables excluded from the matching

**Variables**	**All subjects (*n* = 3849)**		**Marginal matched groups**		**Caliper matched groups**	
	** < 70 years (*n* = 1471)**	** ≥ 70 years (*n* = 2378)**	** < 70 years (*n* = 1471)**	** ≥ 70 years (*n* = 1578)**	** < 70 years (*n* = 1289)**	** ≥ 70 years (*n* = 1289)**
**Sex**						
Women	673 (45.8)	1158 (48.7)	673 (45.8)	773 (49.0)	581 (47.0)	581 (47.0)
Men	798 (54.2)	1220 (51.3)	798 (54.2)	805 (51.0)	655 (53.0)	655 (53.0)
**ASA classification**						
ASA 1–2	1176 (79.9)	1336 (56.2)	1176 (82.1)	1256 (79.6)	1005 (81.3)	1005 (81.3)
ASA 3	249 (16.8)	887 (37.3)	247 (17.2)	302 (19.1)	223 (18.0)	223 (18.0)
ASA 4–5	10 (0.7)	90 (3.8)	10 (0.7)	20 (1.3)	8 (0.6)	8 (0.6)
Missing	38	64	-	-	-	-
**Surgical prioritization**						
Elective	1320 (89.7)	2081 (87.5)	1320 (89.8)	1427 (90.4)	1125 (91.0)	1125 (91.0)
Urgent	150 (10.2)	296 (12.4)	150 (10.2)	151 (9.6)	111 (9.0)	111 (9.0)
Missing	1	1	-	-	-	-
**Clinical stage (cTNM)**						
I	457 (31.1)	902 (37.9)	457 (31.1)	515 (32.6)	396 (32.0)	427 (34.5)
II	303 (20.6)	558 (23.5)	303 (20.6)	365 (23.1)	269 (21.8)	295 (23.9)
III	531 (36.1)	718 (30.2)	531 (36.2)	537 (34.0)	465 (37.6)	408 (33.0)
IV	177 (12.0)	193 (8.1)	177 (12.1)	161 (10.2)	106 (8.6)	106 (8.6)
Missing	3	7	-	-	-	-
**Tumour location**						
Colon	935 (63.6)	1797 (75.6)	935 (63.7)	1046 (66.3)	816 (66.0)	816 (66.0)
Rectum	533 (36.2)	578 (24.3)	533 (36.3)	532 (33.7)	420 (34.0)	420 (34.0)
Missing	3	3	-	-		
**Intraoperative bleeding (ml)**						
0	189 (12.8)	277 (11.6)	189 (13.4)	188 (11.9)	158 (12.8)	158 (12.8)
1–249	854 (58.1)	1377 (57.9)	854 (60.4)	955 (60.5)	780 (63.1)	780 (63.1)
250–499	185 (12.6)	295 (12.4)	185 (13.1)	211 (13.4)	154 (12.5)	154 (12.5)
500–999	101 (6.9)	222 (9.3)	101 (7.1)	157 (9.9)	90 (7.3)	90 (7.3)
> 1000	84 (5.7)	99 (4.2)	84 (5.9)	67 (4.2)	54 (4.4)	54 (4.4)
Missing	58	108	-	-	-	-

The values were expressed as number (%)

*Abbreviations*: *ASA* American Society of Anaesthesiologists, *TNM* tumour node metastasis

### Outcome variables

Univariate analyses on all subjects revealed that older patients had a higher 90-day mortality rate (4.6% vs 1.4%, *MPD* 3.3 [95% *CI* 2.2–4.4], *p* < 0.001) than younger patients. Furthermore, 1-year mortality rate was significantly higher among older patients (10.6% vs 4.6%, *MPD* 5.9 [95% *CI* 4.2–7.6], *p* < 0.001). Regarding the secondary outcome variables, there was a statistically significant increase in surgical complications in the younger cohort (Table 2) but no significant differences regarding other complications, ICU care, reoperations, or readmissions. These differences remained in univariate analyses of the matched groups (Table 2).

**Table 2:** Univariate analyses of mortality and morbidity following resection surgery, marginal matched, and caliper matched groups. Multivariable analyses of mortality and morbidity after resection surgery, all subjects. stratified by age

**Variables**	**Marginal matched groups**				**Caliper matched groups**				**Multivariable analyses — all subjects**
	** < 70 years (*n* = 1471)**	** ≥ 70 years (*n* = 1578)**	**MPD (95% CI),* p*-value**	**Effect size**	** < 70 years (*n* = 1236)**	** ≥ 70 years (*n* = 1236)**	**MPD (95% CI), *p*-value**	**Effect size**	**OR (95% CI), *p*-value**
**90-day mortality**									
Yes	20 (1.4)	50 (3.2)	1.8 (0.7–2.9), **0.0011**	0.12	15 (2.1)	33 (2.7)	1.5 (0.3–2.6), **0.012**	0.11	2.12 (1.26–3.58), **0.005**
**Surgical complications**									
Yes	255 (17.3)	228 (14.4)	− 2.9 (− 5.6–( −)0.2), **0.033**	0.08	215 (17.4)	167 (13.5)	− 3.9 (− 6.8–( −)1.0), **0.009**	0.11	0.84 (0.69–1.03), 0.096
**Complications requiring treatment**									
Yes	518 (35.4)	538 (34.3)	− 1.1 (− 4.6–2.3), *P* 0.54	0.02	418 (34.0)	420 (34.2)	0.1 (− 3.7–4.0), 0.98	0.00	0.98 (0.84–1.15), 0.81
**ICU care**									
Yes	73 (5.0)	91 (5.8)	0.8 (− 0.9–2.5), *P* 0.38	0.04	54 (4.4)	56 (4.6)	0.1 (− 1.6–1.9), 0.94	0.01	1.01 (0.73–1.39), 0.97
**Reoperation**									
Yes	129 (8.8)	125 (8.0)	0.9 (− 2.9–1.2), *P* 0.42	0.03	110 (9.0)	98 (8.0)	− 1.0 (− 3.3–1.3), 0.41	0.04	0.95 (0.73–1.23), 0.68
**Readmission**									
Yes	156 (10.7)	137 (8.7)	− 1.9 (− 4.1–0.2) *P* 0.081	0.07	124 (10.1)	109 (8.9)	− 1.2 (− 3.6–1.2), 0.33	0.04	0.81 (0.63–1.03), 0.08
**1-year mortality**									
Yes	68 (4.6)	136 (8.6)	4.0 (2.2–5.8), *P* < **0.001**	0.16	55 (4.4)	96 (7.8)	3.3 (1.4–5.3), **0.0007**	0.14	1.89 (1.38–2.60), < **0.0001**

Variables presented as *n* (%). Univariate analyses were conducted using Fisher’s exact test, values expressed as MPD (95% CI) and effect size (absolute difference in mean SD). Multivariate analyses were made using a multivariable logistic regression model, adjusted for sex, ASA grade, elective vs urgent procedure, TNM stage, tumour location and intra-operative bleeding, values expressed as OR (95% CI)

*Abbreviations*: *ASA* American Society of Anaesthesiologists, *CI* confidence interval, *ICU* intensive care unit, *MPD* mean percent difference, *OR* odds ratio, *SD* standard deviation

In multivariable logistic regression analyses, the dependent variable was adjusted for sex, ASA classification, elective vs urgent surgery, TNM stage, tumour location, and intraoperative bleeding. The increased 90-day and 1-year mortality for the older patients remained in these analyses. There were no significant differences in any of the other secondary outcome variables (Table 2).

**Fig. 2:**
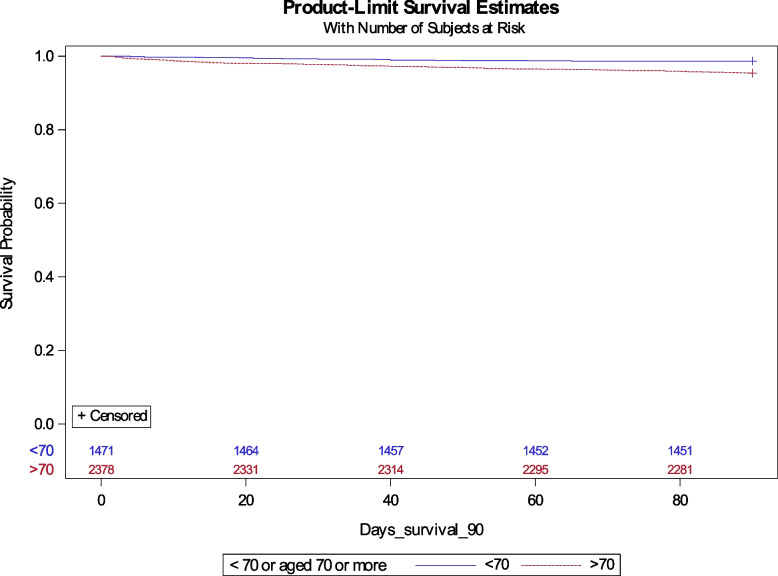
Kaplan–Meier curve of 90-day survival post resection surgery, all subjects, stratified by age

In survival analyses of all subjects, adjusted for all known baseline variables, using the Cox proportional hazard model, higher age was associated with poorer 90-day survival rates (*HR* 2.05 [1.24–3.39], *p* < 0.001). One-year survival was similarly impaired in the older cohort (*HR* 1.71 [1.29–2.29], *p* 0.0002). The 90-day survival analysis of the whole study cohort is visualised by a Kaplan–Meier curve (Fig. 2).

Further, multivariate analyses comparing 90-day post-operative mortality between the two age groups stratified for surgical prioritisation were conducted. These analyses revealed no significant difference in 90-day mortality between the two age groups following urgent resection surgery (*OR* 2.13 [95% *CI* 0.92–4.91], *p* 0.077).

## Discussion

The results from this study confirm that age is a risk factor of increased 90-day and 1-year mortality after CRC resection surgery. The current dataset does not provide information regarding cause of death, but 90-day mortality is assumed to be related to the recent surgical procedure. However, differences in 1-year mortality are more difficult to relate to this specific event. It is not surprising that the elderly cohort has a higher 1-year mortality rate compared to the younger cohort, and in this observational study, we do not have the explanatory variables to establish if this increase is related to the surgical procedure, the cancer diagnosis, or an overall increased mortality rate related to ageing.

In the present study, older patients more frequently underwent urgent resection surgery than younger patients. This is in accordance with previous international studies regarding post-operative mortality and morbidity [5, 6, 12, 17], though in our study there was no statistically significant increase in post-operative mortality after urgent surgery. However, the OR (2.13) for 90-day mortality following urgent resection surgery compared between the two subgroups suggests that there may be an increased risk of post-operative mortality following urgent procedures in the older adults, though our study may have been underpowered to establish this.

Previous work has reported that cancer-specific survival for patients with CRC is not age related [1, 12, 14, 30, 31], though post-operative mortality is increased [6]. This suggests that the immediate post-operative period is crucial, and if elderly patients survive the first period post-surgery, they do not seem to suffer higher risk of cancer-related death in the long term, even if their overall survival is lower compared to younger individuals [1]. Chronological age is of importance but probably not the most important factor to consider when attempting to evaluate risks in the elderly prior to CRC resection surgery. In the future, focus should be on reduction of operative risks in the elderly. As the demographics in the Western world are changing towards an increased life expectancy, colorectal cancer resection surgery in ageing individuals is likely to increase further. Attention must be brought to the fact that the elderly population is a heterogenous group, and that there is a need of identifying high-risk individuals with small margins and elevated risks. 

This is a retrospective study where all analyses were restricted to variables registered in the SCRCR, which implies certain limitations. Although we adjusted for known confounding factors, there is a risk that our results could have been influenced by an unknown factor which we have not considered or did not have knowledge of. An obvious study limitation is the restricted knowledge of comorbidities and functional status of the participants, the only indicator of comorbidities in the registry being ASA grade. This is a rather blunt way of assessing a patients’ medical status, with known limitations, for example inconsistency between anaesthesiologists [32]. Taking age and ASA into account gives information of a patient’s overall health care status, including pre-existing cardiovascular and pulmonary comorbidity, but has limitations in further risk estimation. Another option would be to add a frailty evaluation to enable a more comprehensive assessment of the elderly individual’s risk [33–36]. In other quality registers, such as the Swedish Register of Information and Knowledge about Swedish Heart Intensive Care Admissions (RIKS-HIA), frailty is a mandatory variable [37].

Identifying who is likely to benefit from a treatment is a central part of the surgeons’ decision-making process. There is a risk that these decisions could be made on arbitrary grounds, especially if too much focus is on chronologic age. Using frailty as means of assessing biological age and as a comprehensive estimate of individual resources and deficits can help the practitioner to make well-grounded decisions regarding treatment choices.

Frailty is an independent risk factor for adverse events, also in terms of colorectal cancer surgery [17, 38–42]. Frailty can be seen as a marker of biological age and constitutes a clinical syndrome of reduced reserves and increased vulnerability [34, 35]. It is a topic of research interest in the colorectal cancer community, and our research group is currently conducting a randomised controlled trial regarding frail elderly individuals undergoing elective colorectal cancer surgery [43]. The present study confirms the need for further developments in the treatment of older colorectal cancer patients, a growing part of the population. Future studies should focus on frailty assessments and its correlation to post-operative outcome in older adults, as frailty may be one of the unknown explanatory, risk-predictive, factors contributing to the higher post-operative mortality seen in the older group of our dataset.

## Conclusions

Older adults have increased postoperative mortality following CRC resection surgery, although their cancer-specific survival rates do not differ from younger populations. As the older population is steadily growing and will require surgery for colorectal cancer in an increasing extent, more efficient ways of determining individual risks need to be implemented in clinical praxis.

## Data Availability

The datasets used and analysed during the current study are available from the corresponding author on reasonable request.

## Abbreviations

ASA: American Society of Anaesthesiologists
CI: Confidence interval
CRC: Colorectal cancer
HR: Hazard ratio
ICU: Intensive care unit
MPD: Mean percent difference
OR: Odds ratio
RIKS-HIA: Swedish Register of Information and Knowledge about Swedish Heart Intensive Care Admissions
SCRCR: Swedish Colorectal Cancer Registry
TNM: Tumour nodes metastasis
